# Gastroschisis, a congenital anterior abdominal wall defect: a rare clinical image

**DOI:** 10.11604/pamj.2022.42.298.36378

**Published:** 2022-08-22

**Authors:** Ashna Gledina, Seema Singh

**Affiliations:** 1 Department of Medical Surgical Nursing, Smt. Radhikabai Meghe Memorial College of Nursing, Datta Meghe Institute of Medical Sciences, Sawangi, Wardha, Maharashtra, India

**Keywords:** Gastroschisis, congenital malformation, birth defect, abdominal wall defect

## Image in medicine

Gastroschisis is a relatively rare birth defect in which the baby´s intestines (stomach, large or small intestines) extends outside of the abdominal wall or exit their body from a 2 to 5 cm hole, most often on the right side beside their belly button during fetal development. It occurs in about 1 in every 2,000 babies. We report a case of a large gastroschisis containing intestinal loop. A 29-year-old, primigravida, was referred to the centre for childbirth. Antenatally, at 24^th^ weeks the fetus was diagnosed with a congenital malformation of the anterior abdominal wall. The ultrasound at 30 weeks, confirmed the diagnosis. At 37 plus weeks, she was taken for emergency caesarean section. A male child was born with the confirmation of presence of gastroschisis. Birth weight 3.2 Kg, height 54 cm with appearance, pulse, grimace, activity, and respiration (APGAR) score 8/9. At birth gastroschisis contained intestinal loop. Newborn was referred to neonatal intensive for further management.

**Figure 1:**
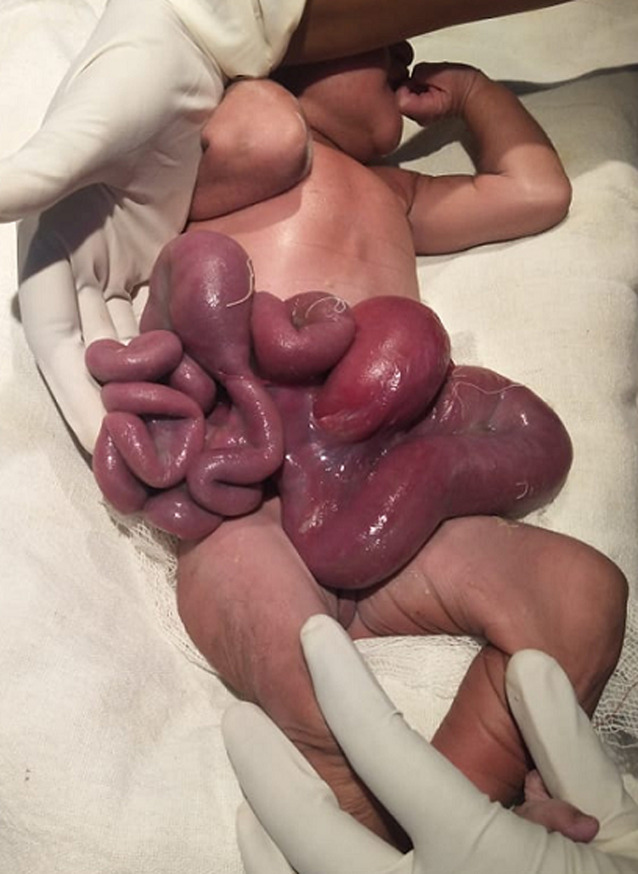
clinical image showing loops of the baby´s intestines (stomach, large and small intestines) extended outside the abdomen wall

